# Ductus Venosus Doppler Flow Velocity after Transplacental and Non-transplacental Amniocentesis during Midtrimester

**DOI:** 10.12669/pjms.305.5065

**Published:** 2014

**Authors:** Burcu Artunc Ulkumen, Halil Gursoy Pala, Yesim Bulbul Baytur, Faik Mumtaz Koyuncu

**Affiliations:** 1 Burcu Artunc Ulkumen, Celal Bayar University School of Medicine, Obstetrics and Gynecology Department, Perinatology Division, Manisa, Turkey.; 2 Halil Gursoy Pala, Celal Bayar University School of Medicine, Obstetrics and Gynecology Department, Perinatology Division, Manisa, Turkey.; 3 Yesim Bulbul Baytur, Celal Bayar University School of Medicine, Obstetrics and Gynecology Department, Perinatology Division, Manisa, Turkey.; 4 Faik Mumtaz Koyuncu, Celal Bayar University School of Medicine, Obstetrics and Gynecology Department, Perinatology Division, Manisa, Turkey.

**Keywords:** Ductus venosus Doppler, Amniocentesis, Mid-cerebral artery Doppler, Uterine artery Doppler, Umbilical artery Doppler

## Abstract

***Objective:*** We aimed to evaluate ductus venosus Doppler waveforms before and after amniocentesis in order to investigate any effect of amniocentesis on fetal myocardial hemodynamics. We also evaluated the umbilical artery, uterine artery and fetal mid-cerebral artery Doppler waveforms in order to investigate any relationship with ductus venosus Doppler changes.

***Methods:*** The study population consisted of 56 singleton pregnancies having genetic amniocentesis. Twenty seven of them had transplacental needle insertion; whereas 29 of them had non-transplacental amniocentesis. Uterine artery, umbilical artery, mid-cerebral artery and ductus venosus pulsatiliy index and resistance index were measured just before and after amniocentesis.

***Results:*** Amniocentesis does not cause any significant changes in fetal ductus venosus Doppler waveforms. There is also no significant changes in uterine artery, umbilical artery, mid-cerebral artery pulsatility and resistance index.

***Conclusion:*** Amniocentesis-whether transplacental or not- does not cause any significant effect on fetal myocardial hemodynamics.

## INTRODUCTION

Genetic amniocentesis (AC) is widely used in obstetric practice. Fetal loss rate does not exceed 0.3% to 1.0%.^1^^-^^3^ Second trimester AS is safe, which is performed at 15 to 20 gestational weeks. The amniotic fluid volume is approximately 125 mL, and then every week, it increases 50 mL until the 28 gestational weeks.^4^ However, it should not be performed before 15 gestational weeks. Before 15 gestational weeks chorion villus sampling (CVS) is safer method compared to early AC due to increased fetal loss rate (7.6% in early AC, 5.9% in CVS) and talipes (RR 4.61; 95% CI: 1.82 to 11.66) in early AC.^5^ Amniotic fluid sampling is not only used for genetic evaluation, it can also be used for diagnosis of metabolic and infectious diseases. AFP levels in amniotic fluid can be also measured for some neural tube defects.^4^

There are a few studies regarding the Doppler velocity waveforms after second trimester AC in order to investigate uteroplacental and fetal circulation.^6^^-^^8^ Ductus venosus is the most accurate tool to interprete both fetal cardiac function and myocardial hemodynamics.^8^ To the best of our knowledge, this study is the first by investigating ductus venosus Doppler velocity forms after AC. Ductus venosus is one of the shunt mechanisms during the intrauterine period. It connects inferior vena cava and intra-abdominal part of the umbilical vein. Ductus venosus directs well-oxygenated blood from the maternal circulation to the right atrium in order to supply mainly the fetal heart and the fetal brain.^9^

Doppler evaluation of the fetal ductus venosus enabled the clinicians too understand many fetal conditions better. The ductus venosus has an important role in the regulation of nutrient partitioning and oxygen supplying in the fetus. Alterations mainly in cardiac afterload and contractility, intravascular volume status or heart rate may significantly impact on the ductus venosus flow velocity waveform.^9^ Accordingly, ductus venosus Doppler is useful in the evaluation and management of conditions that put the fetus at risk for cardiovascular deterioration. From that point of view, we hypothesized that if midtrimester genetic AC had any effect on fetal blood circulation, ductus venosus should be the first in reflecting these changes. So, we aimed to evaluate ductus venosus Doppler waveforms before and after amniocentesis in order to investigate any effect of AC on fetal myocardial hemodynamics.

## METHODS

This study consisted of 56 singleton pregnancies scheduled for genetic AC during midtrimester between April 2013 and February 2014. Local Institutional Ethics Committee approved the study and informed consent was signed by all the participants. The study was conducted prospectively to evaluate the influence of transplacental needle passage on uterine artery (UtA) pulsatility index (PI), resistance index (RI); umbilical artery (UA) PI and RI; ductus venosus (DV) PI and RI; fetal mid-cerebral artery (MCA) PI and RI. Pregnancies with fetal structural abnormality and aneuploidy; multiple pregnancies were not included in the study.

The Doppler velocity waveforms were obtained using Voluson 730 Pro system with a RAB 3.5-MHz array probe (GE Medical Systems, Milwaukee, WI). AC was performed via the same sonographic equipment.

The ultrasound-guided AC was performed using freehand technique. The needle was 20-G in size. The intervention was performed by one of the authors. All the sonographic examination just before and after AC was performed by one of the authors. All patients had only one needle insertion. The sample volume of amniotic volume was approximately equal to the gestational week. 

UA Doppler measurements were obtained in the free loop.^10^^,^^11^ UtA Doppler measurements with transabdominal route were made via color flow mapping to identify the artery crossing the external iliac artery.^10^^,^^12^ Fetal MCA doppler evaluation was made by obtaining a fetal axial section including fetal thalamic nuclei on the scan. Color flow mapping was used to identify the circle of Willis. The measurement was made on the proximal third of the MCA where it is close to its origin in the internal carotid artery. DV is seen by midsagittal plane of the fetal trunk. Color flow mapping helped us to identify the alignment where the high velocity of the vessel can be seen at its narrow entrance.^10^ All Doppler waveforms were calculated only after obtaining three consecutive waveforms.

Statistical analysis was performed by using SPSS v.20 (SPSS Inc., Chicago, IL). The results were expressed in mean ± standard deviation (SD). Student’s t-test for paired variables was used for the intra-group differences before and after AC. Student’s t-test for unpaired variables was used to evaluate the group differences. Spearmen’s correlation analysis was conducted for investigating the relation between Doppler results and gestational week.

## RESULTS

Sixty women participated in the study.^4^ AC results revealing chromosomal abnormalities were excluded from the study. 29 of 56 participants had posterior located placenta and so non-transplacental amniocentesis (NTP). 27 of them had anterior located placenta and so transplacental amniocentesis (TP). Mean maternal age was 29.81±4.43 Indications for amniocentesis were maternal anxiety due to advanced maternal age (n=10), increased risk in first and second trimester screening tests (n=46). 

Mean gestational week was 18.32±1.61 and 17.84±1.82 in TP and NTP group, respectively (p= 0.314). Correlation analysis revealed no significant relationship between gestational week and UA PI, MCA PI, UtA PI and DV PI. The correlation analysis was shown on the Table-I. The Doppler velocity waveforms were shown on Table-II. There was no significant difference in UtA, UA, MCA, DV PI and RI forms between TP and NTP groups before and after AC.

## DISCUSSION

AC is a widely used intervention used in prenatal diagnosis of both chromosomal anomalies and fetal infections.^5^^,^^6^ To the best of our knowledge, our study is the first to evaluate ductus venosus Doppler flow after AC and any correlation between ductus venosus Doppler and other Doppler waveforms in feto-maternal circulation.

**Table-I:** Correlation analysis between gestational week and Doppler PI

***GA***	***UA PI***	***MCA PI***	***DV PI***	***RUtA PI***	***LUtA PI***
r	0.044	-0.056	-0.083	-0.051	-0.194
p	0.757	0.695	0.580	0.728	0.187

RUtA: right uterine artery; LUtA: left uterine artery; PI: pulsatility index;

r: Spearmann’s correlation coefficient.

* GA: gestational age (week); UA: umbilical artery; MCA: mid-cerebral artery; DV:ductus venosus;

**Table-II:** Doppler flow velocity measurements in TP and NTP group before and after AC

	***TP group*** ***(n=27)***		***NTP group*** ***(n=29)***		***p***	
	***Before AC***	***After AC***	***Before AC***	***After AC***	***Before AC***	***After AC***
UA PI	1.750.81	1.840.72	1.500.40	1.620.12	0.188	0.600
UA RI	0.830.18	0.840.23	0.800.14	0.910.21	0.465	0.263
MCA PI	2.170.85	2.851.97	2.230.85	2.761.15	0.769	0.852
MCA RI	0.910.13	0.950.11	0.930.11	0.960.11	0.470	0.941
DV PI	0.910.49	1.280.71	1.050.97	1.930.67	0.535	0.182
DV RI	0.650.33	0.780.48	0.620.38	0.690.31	0.749	0.100
RUtA PI	1.320.54	1.590.17	1.420.78	1.420.62	0.611	0.351
RUtA RI	0.660.14	0.840.13	0.670.13	0.680.15	0.778	0.407
LUtA PI	1.560.74	1.560.87	1.510.72	1.530.69	0.807	0.980
LUtA RI	0.710.14	0.740.29	0.690.13	0.690.15	0.751	0.472

UA: umbilical artery; MCA: mid-cerebral artery; DV: ductus venosus; RUtA: right uterine artery;

LUtA: left uterine artery; PI: pulsatility index; RI: resistance index.

Ductus venosus is one of the shunt mechanisms during fetal life which is quite different from adult life with the aim of bringing more oxygenated blood to the fetus. Ductus venosus shunts most of the left umbilical vein blood flow directly to the inferior vena cava, thus allowing oxygenated blood to bypass the liver and supply the fetal body; mainly fetal heart and brain.^14^

Ductus venosus is the most accurate tool to interprete both fetal cardiac function and myocardial hemodynamics.^10^^,^^15^^,^^16^ There was only one study about the relation between ductus venosus Doppler and amniocentesis in the published literature. Helbig and co-workers evaluated 99 women undergoing genetic amniocentesis. They found no significant change in PI from before (1.07; 0.54–2.42) to after (1.03; 0.51–3.27) AC. They showed also that TP route had no effect on the results.^17^ Similarly, we found no change in ductus venosus PI from before (0.910.49) to after (2.281.01) AC in TP group; also from before (1.050.97) to after (0.930.67) AS in NTP group (p= 0.535 and p= 0.182, respectively).

Haugen et al. suggested that TP needle passage could induce the release of some substances causing vasoconstriction which in turn may have an effect on the peripheric vascular impedance and cardiac hemodynamics in fetus.^6^ They evaluated UA PI in 168 women having AC. They found no significant difference for both TP and NTP group (p=0.31).^6^ Similarly, we showed no change in umbilical artery PI from before (1.750.81) to after (1.840.72) AC in TP group; also from before (1.050.97) to after (2.122.05) AC in NTP group (p= 0.188 and p= 0.263, respectively).

The main limitation of our study was the small sample size. The second limitation was the lack of the data about the maternal anxiety about the invasive procedure in our study. Caliskan et al assessed maternal anxiety levels with the Spielberger State-Trait Anxiety Inventory in 60 pregnancies undergoing genetic AC.^18^ They showed that maternal anxiety levels had effects on the fetal blood flow.^18^ The longer the duration between the decision of the procedure and the sampling, the greater the maternal anxiety.^18^ From that point, we can suggest that our patients must have had low stress level about the AC, because we booked their procedure in one or two days.

As a result, AC –whether TP or not- does not cause any significant effect on fetal myocardial hemodynamics. It is still a safe method for fetus to learn about genetics and infections.

## Authors Contribution

**Burcu ARTUNC ULKUMEN** conceived, designed and did statistical analysis & writing of manuscript.

**Halil Gursoy PALA** was involved in data collection, interpreting the data and manuscript editing.

**Yesim BULBUL BAYTUR and Faik Mumtaz KOYUNCU** did review and gave final approval of manuscript.
